# Stabilisation of Nanosilver Supramolecular Hydrogels with Trisodium Citrate

**DOI:** 10.3390/molecules30071613

**Published:** 2025-04-04

**Authors:** Joanna Kowalczuk, Oleg M. Demchuk, Mariusz Borkowski, Michał Bielejewski

**Affiliations:** 1 Institute of Molecular Physics, Polish Academy of Sciences, M. Smoluchowskiego 17, 60-179 Poznań, Poland; michal.bielejewski@ifmpan.poznan.pl; 2 Faculty of Medicine, The John Paul II Catholic University of Lublin, 20-708 Lublin, Poland; 3 Jerzy Haber Institute of Catalysis and Surface Chemistry, Polish Academy of Sciences, Niezapominajek 8, 30-239 Kraków, Poland; mariusz.borkowski.edu@gmail.com

**Keywords:** *N*-lauroyl-l-alanine, C_12_Ala, AgNPs, TSC, hydrogel, NMR, TGA, DSC

## Abstract

Designing supramolecular gelators with targeted properties is very difficult and mainly relies on structural modifications of known gelator molecules. However, very often, even minor modifications can result in the complete loss of gelation capabilities. In the present work, we have studied the influence and role of the silver nanoparticles (AgNPs) and trisodium citrate (TSC) additives on the self-assembly process of alanine derivative gelator (C_12_Ala) and intermolecular interactions resulting in hydrogel systems of enhanced stability and sustainability. The effect of phase separation and diversity of supramolecular microstructures of gelator internal matrix on the composition of the investigated tricomponent system was studied thoroughly with thermal analysis methods (TGA/DSC), high-resolution nuclear magnetic resonance spectroscopy (HR-MAS NMR), and polarising optical microscopy (POM). The molecular mechanism of gelation and the interactions responsible for enhanced properties of nanosilver hydrogels was determined and described, indicating the synergistic role of TSC and AgNPs in the self-assembly process.

## 1. Introduction

Soft matter systems like physical gels/hydrogels find broad applications in fuels [1,2], food [3,4] and the pharmaceutical industry [5,6]. The critical parameter for successfully using these materials is their thermal and temporal stability, which depends strongly on the gelator structure and the additives incorporated in the gel. To this day, it remains impossible to predict whether a given molecule has solvent gelling properties or not. Gel research is primarily experimental work. However, sometimes a molecule that does not have solvent-gelling properties on its own acquires this property in the presence of another molecule. An example of such a molecule is *N*-lauroyl-l-alanine (C_12_Ala), which on its own gels water very poorly and does not have the gelling properties of a hydrolate enriched with silver nanoparticles. However, the situation changes radically when the gelator–nanoparticle hydrolate system is enriched with a stabiliser molecule. Non-toxic hydrogels enriched with silver nanoparticles (AgNPs) constitute a group of materials with extremely high pharmaceutical potential [7]. In the case of AgNPs containing gels aside from the metal, the stabilators used in nanoparticle synthesis impact the gel properties. One of the most common stabilators used to synthesise negatively charged AgNPs is trisodium citrate (TSC) [8].

The biological activity of silver nanoparticles has been repeatedly confirmed in the fight against microorganisms (including bacteria, viruses, and fungi) or pathological human cancer cells [9,10,11,12]. The bioactive role of nanoparticles, including silver, depends strongly on their size and can have positive as well as negative effects on living organisms. The toxicology of nanoparticles is complicated, and the mechanism of hemotoxicity of silver nanoparticles, for example, is still not fully understood. It is unclear whether it results from the direct impact of nanoparticles on cells or from the release of silver ions. However, we can conclude that the smaller size of nanoparticles results in their more significant biological activity [13,14,15,16]. Another field where silver nanoparticles can be used is photodynamic cancer therapy (FTD). One of the effects of silver ions interactions with cells is the production of reactive oxygen species, especially their singlet form, which causes damage and cell death. The combination of a photosensitiser with silver nanoparticles leads to an increase in the efficiency of the production of free radicals in cells. One possible way to introduce photosensitisers into diseased areas is through the use of hydrogels [17,18]. Due to the alarming statistics of cancer cases, research on improving anti-cancer therapies is currently a civilisational challenge. We already know that antibiotics supported by nanoparticles improve the effectiveness of drugs, but we need personalised therapies with potentially higher therapeutic efficacy [19,20,21,22].

One of the known systems for delivering active substances to the body are gels [23]. A necessary condition for creating such systems for therapeutic purposes is the use of components (solvents and gelators) that have a neutral effect on the organism, can transform pharmaceuticals into an inactive gel form, and release and activate them at the targeted side. However, finding such a gelator to create non-toxic, durable, and stable systems is difficult. The best candidates are hydrogels, which use water to dissolve and immobilise the drug in the gel phase [24,25]. Recent research on different low-molecular-weight gelators showed that the representative of fatty acid amide C_12_Ala displays the ability to create physical gels with varying types of solvents, including organic solvents, oils, emulsions (oil–water mixture) and water [26,27,28,29,30]. In particular, water is an important solvent that can create gel-based drug carrier systems enriched with gold and silver nanoparticles [9,31].

Although C_12_Ala (LMWGs; low-molecular-weight gelators) has been shown to form hydrogels, it was found that their stability is time-limited. The pure hydrogel C_12_Ala-H_2_O undergoes phase separation within a few hours after gelation. The stable, homogeneous gel (verified by the inverse tube test) disintegrates and begins to resemble the structure of semolina or micellar fluid. While working on the C_12_Ala gel enriched with silver nanoparticles, we noticed that after a few months of storing the C_12_Ala-TSC hydrogel at 5 °C, the solid and liquid phases also began to separate, which resulted in the gravitational outflow of water from the sample. This effect does not bode well for the durability of the resulting gel but did not occur in the case of the gel enriched with nanoparticles. The sample enriched with TSC and AgNPs, tightly closed and stored at 5 °C retained its properties for 5 months. In the publication [9] (see Figure 5a,b), we see photos of upside-down vials with stored gels. The C_12_Ala-TSC-AgNP sample looks homogeneous, but at the bottom of the vial with C_12_Ala-TSC-H_2_0 gel (top of the image), we can see an area where water has accumulated, and the gel does not look homogeneous but rather resembles a “semolina” structure. So, the question arose about the effect of TSC and AgNPs on the internal structure of the gel and how TSC and silver nanoparticles contribute to its longer stability. In this work, the authors attempt to explain this phenomenon.

## 2. Materials and Methods

### 2.1. Hydrogel Preparation

**Figure 1:**
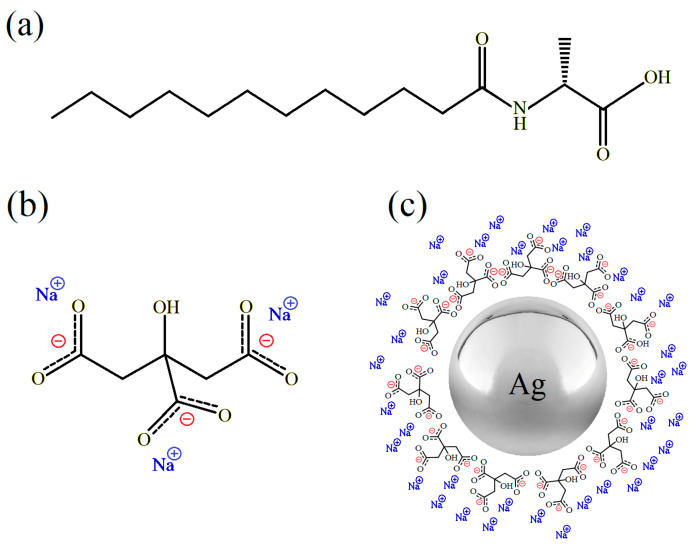
The chemical structure of (**a**) C_12_Ala, (**b**) TSC, and (**c**) illustrative view of negatively charged silver nanoparticles stabilised by TSC.

The investigated samples of hydrogels were prepared according to the following procedures. The C_12_Ala-H_2_O hydrogels were obtained by dissolving 64 mg and 128 mg of the C_12_Ala gelator [32] in 1 mL of deionised water to obtain 6 wt.% and 12 wt.% mixture concentrations, respectively. The dissolution was performed using a circulating water bath at 60 °C for 24 h. After dissolving the gelator molecules, the samples were cooled to room temperature for 12 h. The C_12_Ala-TSC-H_2_O hydrogels were prepared by dissolving 323 mg of TSC in 1 mL of deionised water, resulting in a molar concentration of Cm = 1.25 M. Next, the prepared solution of TSC was mixed with deionised water in a volume ratio of 0.5%/95.5% and used to prepare hydrogel samples with 6 wt.% and 12 wt.% concentrations of gelator. The procedure for dissolving the gelator in the prepared solution and obtaining the gel phase was the same as for the C_12_Ala-H_2_O system. The tube inversion test was performed to confirm no bulk flow. However, only the TSC-enriched sample retained its lack of flow over time. The C_12_Ala-TSC-AgNP hydrogels were prepared by mixing 995 mL of AgNP water suspension and 5 mL of a 1.25 M water solution of TSC with 64 mg and 128 mg of gelator to obtain 6 wt.% and 12 wt.% hydrogels respectively. The silver nanoparticles with an average size of 30 nm (AgNPs) were synthesised as described in [9]. The chemical structures of the used compounds are depicted in Figure 1. The gelator dissolution process was carried out the same way as for other samples. As a result, we obtained three different gel systems: pure hydrogel C_12_Ala-H_2_O (not stable in the concentration of 6 wt.%), hydrogel enriched with TSC (C_12_Ala-TSC-H_2_O), and hydrogel enriched with TSC and silver nanoparticles, AgNPs, (C_12_Ala-TSC-AgNPs). Without the addition of TSC, the C_12_Ala molecule did not gel the nanoparticle solution containing AgNPs.

### 2.2. Differential Scanning Calorimetry (DSC)

Differential scanning calorimetry (DSC) measurements were performed using a Perkin-Elmer DSC4000 instrument (Waltham, MA, USA). The measurements were conducted in a nitrogen atmosphere at a heating/cooling rate of 15 °C/min in the temperature range of 20 to 100 °C. The heating and cooling rate of 15 K/min was slightly higher than the conventional 10 K/min to estimate the safe limit for storing samples in the gel state. The samples weighing approximately 14.4 mg and 12.4 mg for hydrogels with TSC and TSC-AgNPs, respectively, were loaded in hermetic crucibles. Three consecutive cycles of heating and cooling were recorded for each sample. The measurements allowed us to determine the characteristic endothermic and exothermic peaks for melting and gelation temperatures of the self-assembled matrices of the studied hydrogels.

### 2.3. Thermogravimetric Analysis (TGA/DTG)

Thermogravimetric analysis (TGA) was performed on a Perkin-Elmer TGA8000 device. The method allows for recording the mass loss of the sample during its thermal degradation. The measurements were performed under a nitrogen atmosphere for TSC and TSC-AgNP hydrogel samples at 10 °C/min heating rates in the temperature range of 20 to 1000 °C. The results were analysed according to the derivative method (DTG).

### 2.4. NMR Spectroscopy

The ^13^C NMR spectra of the studied samples were recorded using a Bruker Avance III HD NMR spectrometer (Billerica, MA, USA) coupled to an 11.4 T superconducting magnet operating at 125.76 MHz for the carbon Larmour frequency. All experiments were recorded at room temperature using the solid-state technique for high-resolution NMR spectroscopy. The ^13^C spectra were recorded using the ^1^H-^13^C CP-MAS technique to improve the S/N ratio for recorded signals. In the case of the C_12_Ala gelator in the solid state, 2.5 mm zirconia rotors and 20 kHz spinning frequency were used for measurements. To record the ^13^C NMR spectra of TSC solution and hydrogel samples, special 25 μL Kel-F inserts for 4 mm zirconia rotors and 5 kHz of spinning frequency were used. The lower spinning rate and special inserts were used to avoid the risk of mechanically destroying the gel state due to excessive centrifugal force and leakage of the solvent. The contact time during the cross-polarisation sequence was set to 1500 μs, the recycle delay was set to 15 s, and 2048 scans were accumulated, resulting in over 8 h of acquisition time per spectrum. The proton decoupling was achieved with a TPPM sequence at an 80 kHz radiofrequency field.

### 2.5. Microscopic Observation

The images of the gel microstructures were taken on an Olympus BX53 microscope (Tokyo, Japan). The pure, blank, and AgNP-enriched 12 wt.% gels were cast onto microscope slides and covered with a 130 nm thick coverslip. An image of the nanoparticle AgNP solution with the C_12_Ala gelator was also taken for comparison. Images were taken immediately after the samples were placed on the slides.

## 3. Results

### 3.1. Calorimetric Tests (DSC)

**Figure 2:**
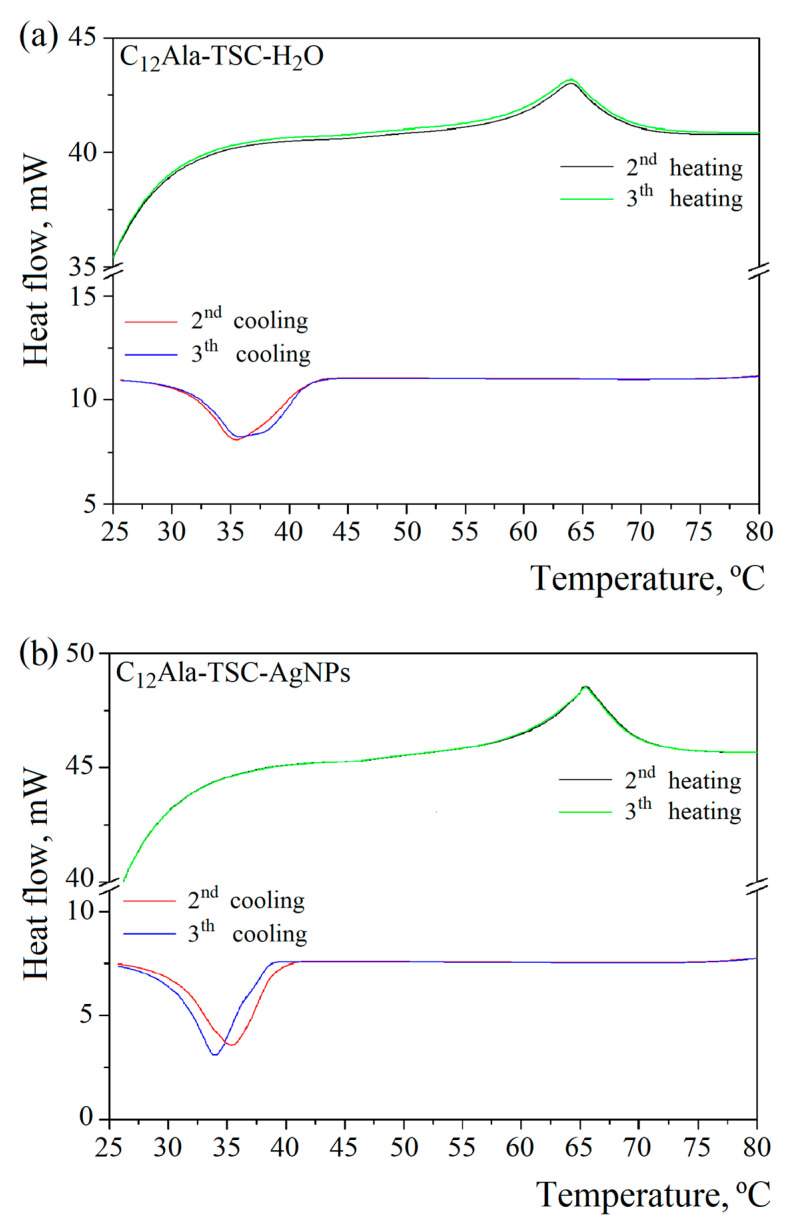
DSC curves of (**a**) C_12_Ala-TSC-H_2_O and (**b**) C_12_Ala-TSC-AgNP gels.

To gain information on thermal events in the blank and silver-enriched sample, DSC scans were performed in the temperature range of 20 to 100 °C. Figure 2a,b show two heating–cooling cycles for blank (C_12_Ala-TSC-H_2_O) and silver-enriched (C_12_Ala-TSC-AgNPs) gels.

The DSC examination revealed two characteristic peaks, gel–sol and gelation, for both samples. The gel–sol points were 64 °C and 65.5 °C for blank and silver-enriched gels, respectively. The gelation temperature varies from 35.5 °C to 34 °C in the second and third cooling cycles for the enriched sample, and for the blank sample, it remains more stable at 35.5 °C. This indicates silver nanoparticles’ influence on the gelator molecules’ organisation in the gelation process. In each subsequent heating and cooling cycle, this process may take place slightly differently.

### 3.2. Thermogravimetric Analysis (TGA + DTG)

**Figure 3:**
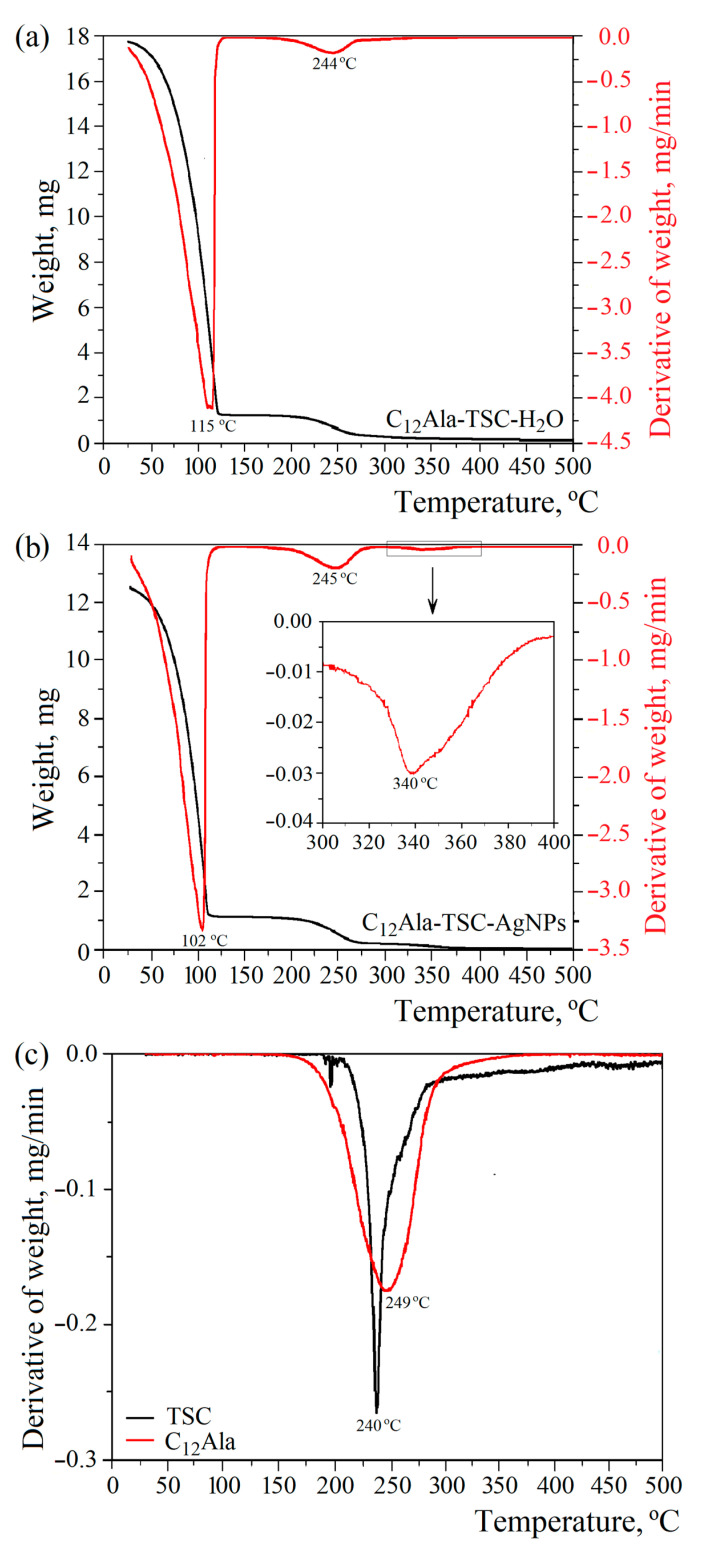

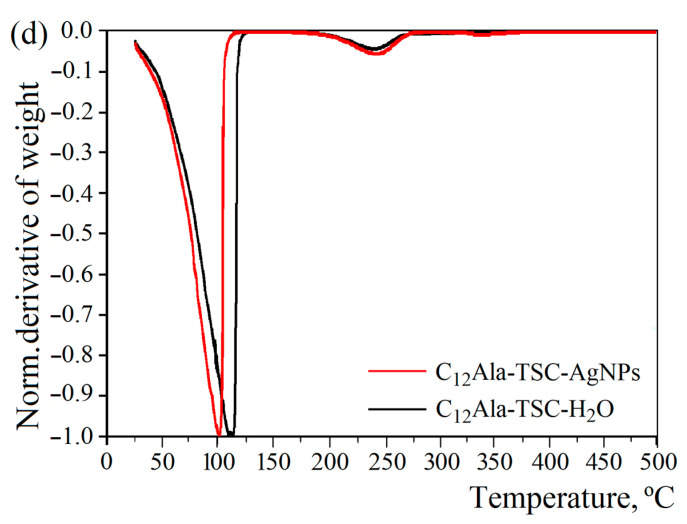
The 10 °C/min TGA and DTG thermograms of (**a**) C_12_Ala-TSC-H_2_O, (**b**) C_12_Ala-TSC-AgNPs, (**c**) DTG curves of TSC and C_12_Ala powder, and (**d**) normalised derivatives of weight loss of gel samples.

Two (for liquid and solid phases) and three (for liquid, solid, and nanoparticles phases) characteristic peaks related to decomposition or evaporation (water) of individual components for the blank (C_12_Ala-TSC-H_2_O) and silver-enriched samples (C_12_Ala-TSC-AgNPs), respectively, are visible on the TGA and DTG curves (Figure 3a,b). Figure 3c represents the DTG curves of TSC and C_12_Ala powder. To compare the degradation temperatures of the individual phases of the systems, Figure 3d shows the normalised mass loss derivatives for both tested samples. The results do not include the pure C_12_Ala-H_2_O hydrogel sample because it disintegrated too quickly for its properties to be compared with the stable samples.

The addition of silver nanoparticles, AgNPs, to the hydrogel of C_12_Ala with TSC influences the temperature water fraction evaporation. The DTG curves (Figure 3b) show a decrease in the inflection point related to the temperature at which the mass loss is the quickest by 13 °C in comparison to the silver-unenriched sample (Figure 3a). The maximum water evaporation rate occurs at 102 °C and 115 °C for the sample with and without silver nanoparticles, respectively. Two possible reasons can cause such behaviour: the intramolecular interactions between the AgNPs and the gelator matrix and the physical nature of the silver nanoparticles themselves. The intramolecular interactions with AgNPs can disturb the mechanism of physical bonding between the gelator molecules, causing a weakening of the gel strength. On the other hand, the physical properties of silver nanoparticles, namely lower heat capacity and higher heat transfer, can cause local heating and additionally weaken the physical bonds between C_12_Ala molecules. Materials with low heat capacity and high thermal conductivity heat up faster and release accumulated energy more quickly. The heat capacity and thermal conductivity of water and silver are 4200 J/kg·K and 0.6 W/m·K and 237 J/kg·K and 429 W/m·K, respectively. Although adding AgNPs decreases the decomposition temperature, it increases the system’s long-term stability at temperatures below 100 °C. The decomposition process observed at temperatures above 300 °C is related to the melting of AgNPs (Figure 3b, highlighted area). The total weight content of silver nanoparticles in the liquid fraction of the gel is 0.44 % (4.4 mg/mL). Uncrushed silver melts at a temperature of 960 °C. However, silver nanoparticles have a melting point of approximately 360 °C (depending on size), so we can conclude that the peaks visible in this temperature region correspond to the decomposition process of silver nanoparticles. The observed peak in the temperature range 175–275 °C is indistinguishable for TSC and C_12_Ala due to the similar decomposition temperature of both materials, as shown in Figure 3c. The maximum rate of water evaporation is 3.32 mg/min at 102 °C and 4.10 mg/min at 115 °C for the enriched and blank samples, respectively.

### 3.3. ^13^C CPMAS NMR

**Figure 4:**
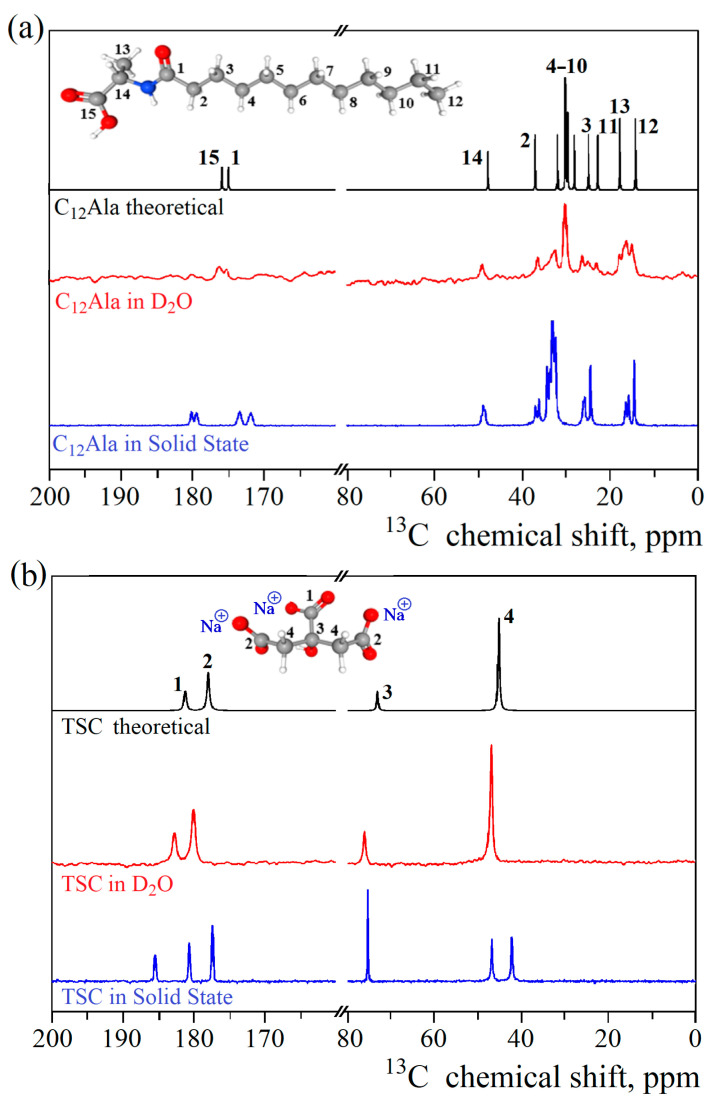

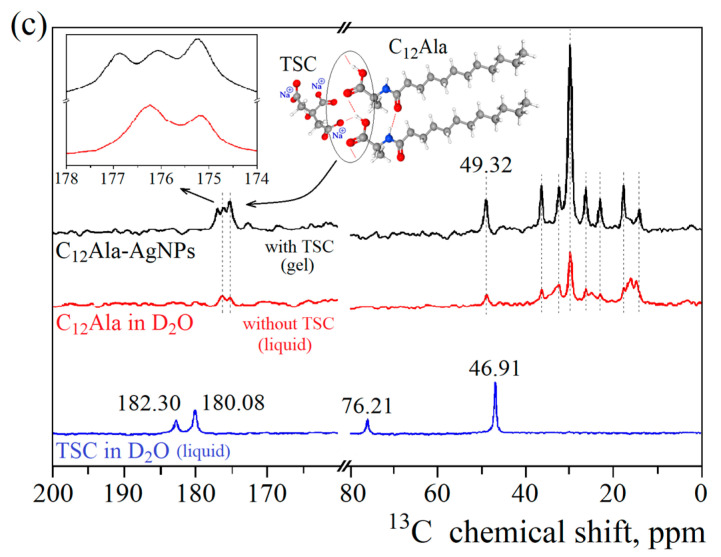
Theoretical and experimental ^13^C CPMAS NMR spectra of solid and D_2_O-dissolved C_12_Ala (**a**) and TSC (**b**), along with experimental spectra of TSC and C_12_Ala dissolved in D_2_O and C_12_Ala-AgNP gel (**c**).

High-resolution NMR techniques were used to investigate the intra- and intermolecular interactions in all studied systems. The theoretical ^13^C NMR spectra were calculated for isolated molecules of TSC and C_12_Ala based on their chemical structures to correctly assign and analyse the obtained experimental results. In Figure 4a,b, the obtained theoretical results are displayed in black, whereas the experimentally recorded spectra in the solid-state are presented in blue. The spectra recorded for C_12_Ala and TSC in the solid state are more complex, as they reveal intermolecular interactions between molecules, leading to splitting and additional resonance lines. Moreover, the static and random orientation of molecules with respect to the magnetic field causes a chemical shift anisotropy and line broadening due to dipole–dipole interactions. The broadening effects can be partially reduced using the magic angle spinning (MAS) technique, allowing the resonance lines to become distinguishable [33,34]. However, changes in chemical shielding caused by non-equivalent interactions between molecules can still be observed. The discrepancy between theoretical and experimental results originates from the fact that theoretical results do not account for the aforementioned effects. However, to test this assumption, we dissolved TSC and C_12_Ala in D_2_O to diminish the intermolecular interactions by dispersing the investigated molecules in a liquid environment, thereby averaging out orientational dipolar interactions through increased rotational and translational molecular dynamics. The experimental ^13^C NMR spectra obtained for diluted liquid samples of TSC and C_12_Ala are presented in Figure 4a,b in red. In the case of TSC, experimental and theoretical results show strong agreement, as expected for a small moleucule soluble in aqueous environments. For C_12_Ala, although the agreement between theoretical and experimental data is much better, especially in the 170–180 ppm range, ir still shows some broadening effects due to residual intermolecular interactions. These observed effects indicate the self-assembly of these molecules, even if gelation does not occur without the addition of TSC, causing slower tumbling of molecules on the NMR timescale due to the formation of structures where diffusion is restricted by interactions. Therefore, the translational and orientational dynamics cannot effectively average out dipole–dipole interactions and the distinct orientation of groups of C_12_Ala molecules with respect to the external magnetic field. Therefore, it is natural to expect that the spectrum in D_2_O will not exactly match the theoretical prediction for monomeric C_12_Ala (in black). On the other hand, to obtain a reasonable S/N ratio, the weight concentration of the gelator molecules in D_2_O was 6% wt.%, which is relatively high, creating a viscous liquid. Sumita and Joykrishna showed that C_12_Ala can experience self-assembly already at a critical aggregation concentration (CAC) of 1.04 mM and at pH 12 or lower [35].

In Figure 4c, the experimental ^13^C NMR spectrum for the C_12_Ala-AgNP gel sample is presented in black, and the ^13^C spectra for liquid samples of TSC and C_12_Ala are given in blue and red, respectively. The observed differences in the gel spectrum originate from the intermolecular interactions established in the self-assembly process during gelation in the presence of TSC molecules. Although we cannot detect the TSC signal directly in the gel sample because of the low concentration, we can see its influence indirectly through the interaction with the COO- groups of the C_12_Ala gelator in the spectral range from 170 to 180 ppm. The observed line at 176 ppm in the liquid sample of C_12_Ala has been split into two lines in the gel sample due to interaction with TSC, mediated by Na+ cation. The inset in Figure 4c shows the splitting and indicates the relative change in the line intensities relative to the line at 175 ppm, which is expected when a single line splits into more components, but the number of interacting spins remains the same. In the 25–10 ppm spectral range, differences between the spectrum of C_12_Ala in the gel and liquid state were detected. This region corresponds to the carbons in the hydrophobic tail of the gelator molecule. Compared to the results for the liquid sample, in the gel sample, the spectrum has narrower lines and is less crowded. This indicates that the aliphatic chain’s carbons exhibit a more uniform chemical environment, resulting in better-defined and uniform chemical shifts. Such a situation occurs when the chains become ordered relative to each other and create a uniform structure, e.g., a well-defined lamellar phase, where all the tails reassemble on the inside and polar groups on the outsides. Based on the obtained experimental results and theoretical spectra, we can conclude that TSC plays a crucial role by triggering the gelation process and interacting with the polar head of the gelator molecules through the sodium cations. The mutual influence of the TSC and AgNP electrostatic interactions on C_12_Ala molecules also causes the ordering of the gelator hydrophobic tails into the lamellar phase.

Figure 4a,b show the ^13^C CPMAS NMR spectra recorded for C_12_Ala and TSC in the solid and D_2_O solution state, together with theoretical spectra calculated for isolated molecules. Figure 4c shows experimental spectra of 1.25 M TSC and 6 wt.% of C_12_Ala dissolved in D_2_O and gel enriched with AgNPs.

### 3.4. Optical Images

**Figure 5:**
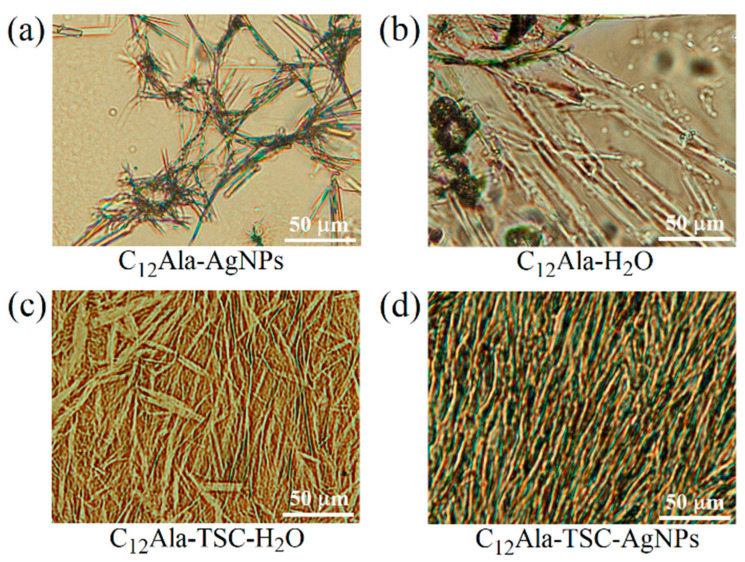
The polarising optical images of 12 wt% C_12_Ala gels: C_12_Ala-AgNPs (**a**), C_12_Ala-H_2_O (**b**), C_12_Ala-TSC-H_2_O (**c**), and C_12_Ala-TSC-AgNPs (**d**).

To investigate the gelation process in the studied systems, images of the microstructures of the samples were recorded using polarising optical microscopy. Drops of the prepared compositions of the studied systems were cast onto amicroscopic plate, covered with glass coverslips, and subjected immediately to observation. The obtained results are presented in Figure 5. The differences between systems with and without TSC were clearly visible to the naked eye and confirmed by microstructure observation (Figure 5a,b vs. Figure 5c,d). The 12 wt.% mixture was used for the tests because 6 wt.% C_12_Ala in water (without TSC) did not form a stable gel with a microstructure that could be recorded in optical microscopy.

The polarising optical images were taken at 20× magnification with a field of view of 275 × 200 μm. The systems containing TSC self-assemble into microstructures that significantly differ from those formed without the addition of TSC. Although the added trisodium citrate is below 0.5 wt.% of the total mass, its influence on the intramolecular interactions between gelator molecules is substantial. The gelator solution with hydrolate C_12_Ala-AgNPs, without TSC, does not exhibit gelling properties. In microscopic images, we can see clusters of gelator fibres surrounded by large pools of water (Figure 5a). In the case of the hydrogels without TSC, such as C_12_Ala-H_2_O (Figure 5b), the 12 wt.% of gelator molecules create rod-like aggregates that are loosely packed, resulting in significant areas where no aggregates are present. The small number of junction points between individual aggregates indicates the low thermal stability of the hydrogels created by the C_12_Ala gelator alone. In the absence of AgNPs, the aggregates of C_12_Ala stabilised by TSC create a dense mesh of rod-like aggregates of different thicknesses, lengths, and dendrimer-like shapes (Figure 5c). With the addition of silver nanoparticles to the systems, the interactions during the self-assembly process upon gelation change, leading to the creation of clusters of aggregates (Figure 5d). The shape of individual gelator aggregates also changes, assembling into thick, short, and straight rod-like shapes similar to the crystallites that create piles of random orientation. These piles can overlap and create regions of highly dense gelator aggregates and large free spaces in between. To explain such a change in aggregation pattern, we assume that the dispersed AgNPs become the nucleation centres for the aggregation of gelator molecules. The most crucial change in the microstructure of the created hydrogels is observed upon the addition of TSC to systems, both in the presence and absence of AgNPs. Such a matrix contains many more junction points and interactions between aggregates, leading to higher thermal and time stability for the hydrogel. Similar to systems without TSC, adding silver nanoparticles to hydrogels containing TSC influences individual aggregates’ shape and the created matrix’s self-assembly pattern. The microstructure of C_12_Ala-TSC-AgNP hydrogels consists of thin, long fibres of dendrimer-like shapes showing spatial ordering. The aggregates have a uniform size and form, indicating the role of AgNPs in the systems at the stage of aggregate formation and the supramolecular self-assembly process. A more uniform matrix in C_12_Ala-TSC-AgNP hydrogels results in higher durability for the systems exposed to temperature and time. Very similar results were obtained in SEM images for 5 wt.% gels with and without AgNPs. In Figure 5a,b, in the publication [9], we can clearly see differences in the structure of the internal gel matrix.

## 4. Discussion

**Figure 6:**
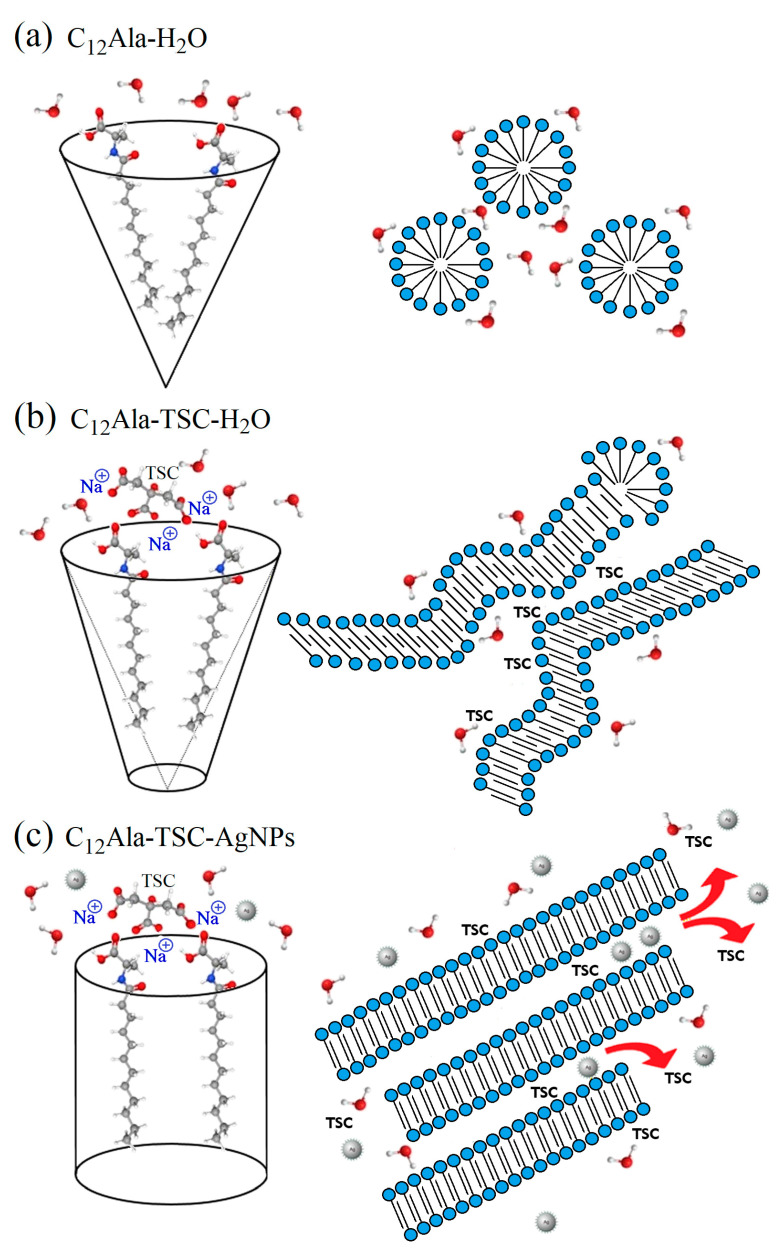
The simplified diagram of the interaction between neighbouring gelator molecules in different conditions: C_12_Ala in water (**a**), C_12_Ala with TSC in water (**b**), and C_12_Ala with TSC in water solution of AgNPs (**c**).

C_12_Ala is an amphiphilic compound with a topological Polar Surface Area (PSA) equal to 66.4 Å^2^ [32] and amino-acid-based surfactants [36] that has a hydrophobic hydrocarbon chain ending with a polar hydrophilic amine and carboxyl groups, so we can expect that, when in contact with polar solutions (e.g., water), it behaves in a characteristic way [37,38,39,40]. Figure 6 shows a simplified diagram of the interaction between neighbouring gelator molecules in different solvent polar conditions. The area of interaction of two neighbouring gelator molecules resembles a cone, with the hydrophobic part of the molecule at the top. C_12_Ala, when in contact with water, forms micelles inside which the hydrophobic parts of the molecules are enclosed (see Figure 6a). Micelles form a colloidal system similar to lysols or emulsions, depending on the degree of dispersion. The addition of TSC to the C_12_Ala–H_2_O solvent results in a lower polarity of gelator molecules; the area of interaction of two neighbouring gelator molecules then resembles a truncated cone, and the mutual interaction of the hydrophobic parts of the molecules decreases. In the tested blank and silver-enriched samples, there is one TSC molecule for approximately 13 C_12_Ala molecules. This is sufficient to change the geometry of the mutual arrangement of the gelator particles. The micelle changes into a lamella system due to the interaction of TSC with C_12_Ala (see Figure 6b), as indicated by the change in shape and the shift of the ^13^C NMR peaks in the range of 170–180 ppm towards higher values, which is visible in the enlarged range in Figure 4c. The polar part of the gelator and silver nanoparticles chelated by TSC has a negative electric charge. Due to the Coulomb interaction, in the silver-enriched sample, C_12_Ala-TSC-AgNPs, the nanoparticles have the ability to organise lamellar fibres into thicker layers, which are held together by their interaction with positively charged sodium ions. The lamella fibres change into a lamella layer system by interacting with AgNPs with C_12_Ala (see Figure 6c). As a result, the gel system is expected to have longer, thicker, and more ordered solid fibrer. It should be remembered that the figure shows a schematic interaction of gelator molecules in the absence or presence of stabilisers such as TSC and AgNPs at CMC (critical micelles concentration—Figure 6a) and CAC (critical assembly concentration—Figure 6b,c). In a real system, the ratio of micelles, lamellae, and lamellar layers depends on the concentration of individual components in the sample, such as the gelator, stabiliser, and nanoparticles.

Now we can understand why, without a small addition of TSC, the C_12_Ala gelator does not gel in the AgNP solution. Adding negatively charged AgNPs to the C_12_Ala hydrogel causes structural changes in the gel. The AgNPs separate C_12_Ala micelles from each other through electrical interaction. A pure hydrogel is formed by short lamellas and micelles, which form gelator clusters or single thin fibres dispersed in water. The disintegration of the pure hydrogel is visible shortly after gelation. In a short time (a few hours), the gelled sample changes into a structure resembling a “micellar fluid”, suggesting an excess of micelles over lamellae in the system. The gelator aggregates precipitate from the gel, and the sample begins to flow. Still, this process is slower than that of a mixture of containing AgNP hydrolate combined only with the gelator C_12_Ala without the participation of the TSC stabiliser. Adding negatively charged silver particles to the system causes an excess micelles of to form relative to the lamellas due to the Coulomb interaction with AgNPs. The fibres break into smaller, unconnected structures. A larger share of weakly interacting micelles in the sample and the separation of gelator fibres make it impossible for the system to gel. As a result, pure hydrogel without added TSC gels only for a short time, whereas the gel enriched with AgNPs without added TSC does not gel at all. This process is visible in Figure 5a,b. In a pure gel (Figure 5b), large pools of liquid are held together by interconnected gelator chains. Still, the Coulomb interaction with negatively charged AgNPs causes the separation of the gelator in the liquid phase (Figure 5a), and the gelation process becomes impossible. The addition of TSC causes an excess of lamellas over the micelles in this system, and the gel becomes more stable, creating short and twisted or unordered fibres in a rigid gel matrix, as seen in Figure 5c for the 12 wt.% gel and Figure 5a at [9] for the 5 wt.% gel. The appearance of lamellar layers also explains the change in the thermal decay rate of the system enriched with nanoparticles in the TGA measurement compared to the blank system. The temperature ranges and amplitudes of thermal degradation of the individual phases of the systems are always larger for samples enriched with nanoparticles than for the blank samples. Heat transfer through thick and long lamella layers occurs slower than in the case of thin and short fibres found in the blank sample. The fluctuation in the temperature of the silver-enriched sample from 33 °C to 36 °C in the DSC measurement in subsequent cooling cycles also becomes understandable. The mutual arrangement of C_12_Ala lamellae due to interaction with AgNPs and the formation of lamella layers may differ slightly in each heating and cooling cycle.

In the f C_12_Ala hydrogel system enriched with silver nanoparticles, AgNPs, the addition of TSC molecules counteracts the electrostatic interaction between the gelator fibres and silver nanoparticles, thereby enabling gelation and maintaining the system’s stability.

## 5. Conclusions

The conducted research allowed us to answer the question about the mechanism of gelation and the intramolecular interactions between the liquid and solid phases in the pure hydrogel of C_12_Ala, the hydrogel of C_12_Ala-TSC, and the silver-enriched hydrogel of C_12_Ala-TSC-AgNPs. The addition of TSC to water, which interacts with the polar part of the gelator molecule and affects its polarity, changes the forming gel matrix’s internal structure, and increases its stability. The C_12_Ala hydrogel, with the addition of a 1.25M TSC water solution, exhibits greater thermal and time stability compared to the pure hydrogel due to the increased number of junction points between individual LMWG aggregates composing the internal matrix. A similar effect was observed in the system containing AgNPs. On the other hand, the presence of only silver nanoparticles in the hydrogel composition without TSC molecules causes the disintegration of the internal structure due to electrostatic interactions between the negatively charged nanoparticles and the micelles formed by the gelator molecules, preventing them from combining into a stable structure. In C_12_Ala-TSC-AgNP hydrogels, the mutual interactions between TSC and AgNPs decrease the negative electrostatic influence of nanoparticles on the LMWG self-assembly process and lead to higher ordering of the created fibrilar aggregates, which increases the homogeneity and strength of the internal matrix. Consequently, we have obtained thermally and long-term stable hydrogels with a homogenous structure and AgNP distribution, improving the potential textural and organoleptic properties of potential pharmaceutical or cosmetic products. The identified role of the TSC molecules in shielding the gelator molecules from electrostatic forces that disturb the self-assembly process introduced by metallic nanoparticles paves the way for the preparation of hydrogel products enriched with other types of nanoparticles.

## Data Availability

The raw data supporting the conclusions of this article will be made available by the authors on request.
